# Immune response stability to the SARS-CoV-2 mRNA vaccine booster is influenced by differential splicing of *HLA* genes

**DOI:** 10.1038/s41598-024-59259-1

**Published:** 2024-04-18

**Authors:** Cíntia Barros Santos-Rebouças, Cristina dos Santos Ferreira, Jeane de Souza Nogueira, Otávio José Brustolini, Luiz Gonzaga Paula de Almeida, Alexandra Lehmkuhl Gerber, Ana Paula de Campos Guimarães, Rafael Mina Piergiorge, Cláudio José Struchiner, Luís Cristóvão Porto, Ana Tereza Ribeiro de Vasconcelos

**Affiliations:** 1 Department of Genetics, Institute of Biology Roberto Alcantara Gomes, Rio de Janeiro State University, Rio de Janeiro, Brazil; 2 Bioinformatics Laboratory-LABINFO, National Laboratory of Scientific Computation LNCC/MCTIC, Getúlio Vargas, Av., 333, Quitandinha, Petrópolis, Rio de Janeiro 25651‑075 Brazil; 3 Histocompatibility and Cryopreservation Laboratory, Rio de Janeiro State University, Rio de Janeiro, Brazil; 4 School of Applied Mathematics, Getúlio Vargas Foundation, Rio de Janeiro, Brazil; 5 Social Medicine Institute Hesio Cordeiro, Rio de Janeiro State University, Rio de Janeiro, Brazil

**Keywords:** SARS-CoV-2, Vaccine response, Whole blood transcriptome, Immune response variability, Admixed population, Human leukocyte antigen, Computational biology and bioinformatics, Genetics, Immunology, Molecular biology

## Abstract

Many molecular mechanisms that lead to the host antibody response to COVID-19 vaccines remain largely unknown. In this study, we used serum antibody detection combined with whole blood RNA-based transcriptome analysis to investigate variability in vaccine response in healthy recipients of a booster (third) dose schedule of the mRNA BNT162b2 vaccine against COVID-19. The cohort was divided into two groups: (1) low-stable individuals, with antibody concentration anti-SARS-CoV IgG S1 below 0.4 percentile at 180 days after boosting vaccination; and (2) high-stable individuals, with antibody values greater than 0.6 percentile of the range in the same period (median 9525 [185–80,000] AU/mL). Differential gene expression, expressed single nucleotide variants and insertions/deletions, differential splicing events, and allelic imbalance were explored to broaden our understanding of the immune response sustenance. Our analysis revealed a differential expression of genes with immunological functions in individuals with low antibody titers, compared to those with higher antibody titers, underscoring the fundamental importance of the innate immune response for boosting immunity. Our findings also provide new insights into the determinants of the immune response variability to the SARS-CoV-2 mRNA vaccine booster, highlighting the significance of differential splicing regulatory mechanisms, mainly concerning *HLA* alleles, in delineating vaccine immunogenicity.

## Introduction

The coronavirus disease 2019 (COVID-19) pandemic, caused by the severe acute respiratory syndrome coronavirus 2 (SARS-CoV-2), posed an unprecedented burden upon global healthcare systems with concurrently substantial economic disruptions, as evidenced by prolonged lockdown measures^1^. COVID-19 vaccines were developed, tested, and approved in record time, substantially reducing the number of cases, hospitalizations, and deaths worldwide^2^. This success, particularly in regions with high vaccination coverage, thereby ratifies that efficacious vaccination approaches harbor the potential to deal with emerging viruses.

SARS-CoV-2 vaccines encompass a broad spectrum of approaches, including inactivated viral particles, live attenuated vaccines, viral vectors encoding the viral spike protein, mRNA constructs, and adjuvanted spike protein subunits^3^. Nonetheless, despite the administration of more than six billion doses of COVID-19 vaccines, the high effectiveness of the COVID-19 vaccines reaching up to 95%^4^, and the fact that the latest viral variants exhibit reduced lethality among immunized individuals, SARS-CoV-2 transmission persisted even at a low speed. Besides, many COVID-19 cases occur in vaccinated individuals^5^. Escape from the immune response or a suboptimal immune response can partially contribute to virus spread, favoring new variants' emergence. However, a comprehensive understanding of the factors underlying differentiated host immune responses to COVID-19 vaccines, even among immunocompetent individuals, is still in progress.

Consistent variations in vaccine immunogenicity are a recurring phenomenon for different viruses. One pivotal aspect requiring thorough characterization concerns host differences in sustaining immunogenicity elicited by vaccines over time and the identification of genetic drivers influencing the immune response to COVID-19 vaccines has been the focus of several studies. Indeed, investigations into the spectral individual variation in functional immune response have revealed a notable genetic connection with vaccination response. Twin studies have provided insights by highlighting that monozygotic twins exhibit lesser variability than dizygotic twins in their vaccine-induced responses under controlled environmental conditions^6^. Furthermore, the host's genetic background, primarily characterized by single nucleotide variants (SNVs) in genes encoding human leukocyte antigen (*HLA*) classes I and II, cytokines, cytokine receptors, and genes involved in innate immune response (e.g., Toll-like receptors), can partly elucidate the interindividual variability in the immune response to vaccines, such as measles and rubella^7–12^, hepatitis B^7,13–15^, influenza^16^, smallpox^17^, or *Bacillus anthracis*^18^. Besides, different ethnic groups living in the same geographical location exhibit diverse immune responses to vaccination or antibodies’ decline, suggesting a genetic modulatory influence in the dynamics of vaccine-induced responses^19^.

Additional factors influencing immune response variability include intrinsic host issues (e.g. age, gender, comorbidities, body mass index, micronutrients, microbiota, preexisting immunity) and extrinsic elements (e.g. smoking, alcohol consumption, exercise, psychological stress, and toxins exposure)^19^. Furthermore, compelling evidence indicates the existence of ethnic diversity in vaccine-induced antibody levels^20^. Thus, efforts directed towards the comprehensive understanding of the wide-ranging spectrum of immune responses observed among healthy individuals and the intricate mechanisms underlying this variability may help to develop more effective immunogenic vaccines and overwhelm vaccine failures. Indeed, high-throughput methodologies, such as RNA-based transcriptome analyses, can be gold standard tools to explore this landscape, since they reflect both intrinsic and extrinsic factors affecting humoral, cellular, innate, cytokine, and adaptive immune responses^21^. Transcriptomic analysis in blood, an accessible tissue reflective of immune system dynamics^22^, can be employed to discern potential signatures of vaccine-induced responses or to characterize their minor expression or complete absence in less responsive individuals.

On a global scale, Brazil emerged as a prominent hub for SARS-CoV-2 spread during pandemics, marked by elevated counts of both cases and deaths^23^ (https://coronavirus.jhu.edu/map.html). Conversely, it boasts a robust immunization program, resulting in significant adherence to SARS-CoV-2 vaccination compared to many other countries. Among the COVID-19 vaccines distributed in Brazil, CoronaVac (Sinovac), a whole inactivated virus vaccine, received regulatory approval from the Brazilian Health Regulatory Agency (ANVISA) in January 2021 and then spread as one of the most globally employed vaccines. The Covishield vaccine was the second vaccine to be administered in the country. It was developed by the University of Oxford in partnership with the pharmaceutical company AstraZeneca, using the modified chimpanzee adenovirus ChAdOx1 as a vector. Subsequently, in response to the emergence of several Variants of Concern (VOCs), notably the Delta (B.1.617.2) and Omicron variants, the Brazilian government introduced the administration of a booster dose of the mRNA BNT162b2 vaccine (BioNTech/Pfizer) to those who had completed the primary vaccination schedule at least six months earlier, aiming to reinforce immune protection against COVID-19^24,25^.

SARS-CoV-2 BNT162b2 vaccine (Pfizer-Biontech) is an mRNA vaccine engineered with lipid nanoparticles and nucleoside modifications, designed to encode a full-length, prefusion-stabilized SARS-CoV-2 spike protein anchored within the viral membrane. It has demonstrated safety and efficacy in preventing COVID-19^26^. Recently, an observational retrospective investigation was conducted to assess the antibody response at intervals of 120 and 180 days after the BNT162b2 vaccine in 1.115 subjects, evidencing that the second dose of this vaccine allows a satisfactory antibody response^27^. Furthermore, booster vaccination enables higher protection against SARS-CoV-2 variants than is achieved with a primary series of vaccination, although antibody titers naturally decrease over time, requiring additional boosting^28,29^. However, studies investigating the impact of genetic regulation of the immune response to the BNT162b2 vaccine and the antibodies' stability have been few and ethnically restricted.

To better understand SARS-CoV-2 vaccine-induced immunogenicity and why some individuals sustain antibody titers after receiving the booster dose better than others, we analyzed the antibody response elicited by the BNT162b2 vaccine. We also carried out bulk RNA-based transcriptome analysis from whole blood 180 days after vaccination boosting. Additionally, our systematic bioinformatic analysis showed a potential role for innate immune regulatory mechanisms in maintaining humoral response after vaccination boosting. Besides, we found high correlations between differential alternative splicing events and vaccine response, mainly concerning *HLA* genes. These genes hold the potential to serve as predictors of vaccine response, highlighting the valuable role of molecular profiling in improving the accuracy of vaccine response prediction.

## Methods

### Study participants and sample collection

Within the context of the Brazilian COVID-19 vaccination campaign, 5,345 individuals from an ethically mixed Brazilian population from Rio de Janeiro (Brazil) were recruited at Rio de Janeiro State University for SARS-CoV-2 IgG evaluation (data not published).

**Figure 1:**
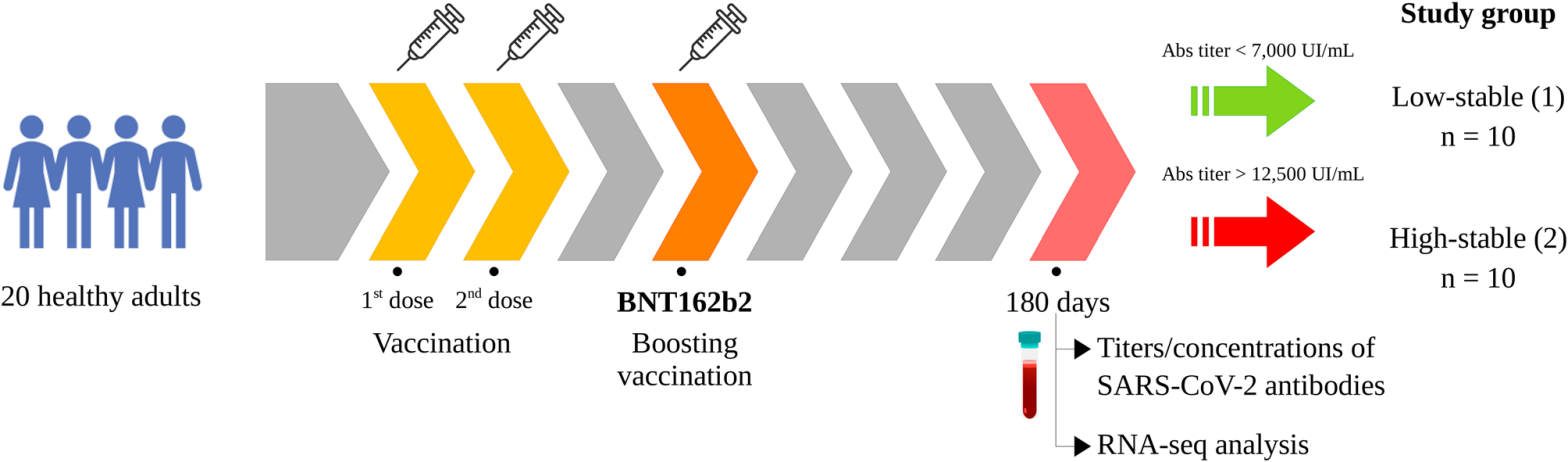
An illustrative representation of experimental design: vaccine characteristics, temporal blood sampling points, and discriminatory antibody profiles for categorizing investigated cohorts.

The primary vaccination schedule in both groups was accomplished by Coronavac (Sinovac) or Covishield (AstraZeneca). Between April and July 2022, twenty healthy adult volunteers’ recipients of a booster (third dose) regimen for the SARS-CoV-2 BNT162b2 vaccine were selected for further evaluation on day 180 after the boosting vaccination (IgG S1 median [min–max] AU/mL, Sinovac: 10,797 [283–80,000], n = 348 and AstraZeneca: 8919 [185–80,000], n = 448). Individuals with systemic conditions, such as cancer, diabetes, and obesity, were not included in this study (Table 1). Within this cohort, individuals were classified into two age- and sex-matched groups of ten individuals, according to the titers/concentrations of SARS-CoV-2 antibodies low-stable (1), in which individuals had antibodies titers/concentrations 180 days less than 7000 AU/mL (percentile < 0.4) after boosting vaccination; and high-stable (2), with antibodies titers compatible with the population in the same period (over 12,800 AU/mL—percentile > 0.6) (Fig. 1).

**Table 1:** Summary features of participants.

Code number	Study group	Gender	Age (years)	Reported ethnicity	Previous reported COVID-19	Primary vaccination	IgGS1 (UI/mL)
953418	1	M	60	Black	Yes (8 months before booster)	AZ	730
2318275	1	F	62	Caucasian	No	AZ	834
2315722	1	F	65	Caucasian	No	AZ	1428
2260337	1	F	35	Caucasian	No	SV	2291
1767816	1	F	47	Mixed	Yes (18 months before booster)	SV	4235
1948860	1	F	41	Mixed	No	SV	4375
1293289	1	F	66	Mixed	No	AZ	4815
2302804	1	M	65	Caucasian	No	SV	6546
1830073	1	F	31	Caucasian	Yes (19 months before booster)	SV	6562
1714685	1	M	65	Mixed	No	SV	6943
2323629	2	F	59	Caucasian	Yes (12 months before booster)	AZ	18,092
2300965	2	F	29	Mixed	No (positive IgG)	SV	12,756
2271208	2	F	44	Mixed	Yes (14 months before booster)	AZ	12,831
2258909	2	F	26	Caucasian	No	SV	27,200
2233454	2	F	54	Caucasian	No	SV	22,442
2304787	2	M	60	Mixed	No	AZ	30,759
2258,498	2	F	31	Mixed	Yes (21 months before booster)	AZ	16,537
881852	2	M	58	Mixed	No	AZ	14,364
2269145	2	M	54	Mixed	No	SV	18,095
2264883	2	F	23	Mixed	Yes (14 months before booster)	SV	17,012

Group 1: individuals with a less stable response according to IgGS1; Group 2: individuals with a more stable response according to IgGS1; M: male; F: female; SV: Coronavac (Sinovac); AZ: Covishield (AstraZeneca); IgGS1: IgG antibodies directed against the nucleoprotein or spike protein of SARS-CoV-2.

The collection of serum specimens for SARS-CoV-2 antibodies analysis and whole blood samples for RNA analysis occurred at the same time, 180 days after the BNT162b2 booster dose. Serum samples for antibody analysis were collected employing vacutainer clot-activator tubes (BD Biosciences, San Jose, CA). Peripheral blood samples for RNA-seq analysis were collected using Tempus™ Blood RNA Tube (Thermo Fisher Inc.).

### Serological assessment of SARS-CoV-2 IgG antibodies

Serum specimens underwent comprehensive analysis to discern the qualitative and quantitative presence of IgG antibodies directed against the nucleoprotein or spike protein of SARS-CoV-2. The automated immunoassay technology was employed, specifically the SARS-CoV-2 IgG and SARS-CoV-2 IgG II Quant assays by Abbott Diagnostics (Abbott Parl, IL). The technique harnessed paramagnetic microparticles coated with either the nucleoprotein or the receptor binding domain (RBD) situated within the S1 subunit of the spike protein. This analytical protocol utilized chemiluminescence (CMIA) processes and outcome measurements are reported as an index for nucleoprotein detection or as arbitrary units per mL (AU/mL) for the spike protein, with a diagnostic threshold set above 1.4 for nucleoprotein reactivity and above 50.0 AU/mL for spike protein reactivity.

### RNA extraction and whole blood transcriptome

Total RNA was extracted from whole peripheral blood samples stored in Tempus™ Blood RNA Tubes using Tempus™ Spin RNA Isolation Kit (Thermo Fisher Scientific, San Jose, CA, USA) followed by treatment with TURBO DNA-free™ Kit (Thermo Fisher Scientific, San Jose, CA, USA). Qubit 2.0 Fluorometer with the Qubit RNA Assay Kit (Life Technologies, Carlsbad, CA, USA), and TapeStation 2200 (Agilent Technologies, Santa Clara, CA, USA) were employed to assess the concentration, purity, and integrity of the RNA samples. Only those samples with an RNA integrity number (RIN) greater than 7.0 were used for subsequent analysis. An average of 0.5 µg of total RNA was utilized for library construction through the Illumina Stranded Total RNA Prep, and rRNA depletion was performed with Ribo-Zero Plus (Illumina, San Diego, CA, USA), following the manufacturer's guidelines. RNA-Seq libraries were sequenced in an Illumina NextSeq 550 platform (75 bp paired-end reads). The quality of sequenced reads was assessed with FastQC^30^, and trimming was carried out using BBDuk (https://sourceforge.net/projects/bbmap). STAR tool version 2.7 was employed for the alignment of the reads onto the human genome reference (GRCh38.p14)^31^. The sequencing metrics were assessed with RNA-SeQC software^32^.

### Identification of differentially expressed genes, alternative splicing events, and functional enrichment

We compared the groups exhibiting low and high stability concentrations of antibodies to identify genes with baseline expression that could potentially serve as predictors for the BNT162b2 vaccination outcome. Differential gene expression analysis was performed through the DEGRE package for R^33^, which identifies Differentially Expressed Genes (DEGs) in a pairwise manner and considers the insertion of the individuals' age as random effects in the experimental design. It also has a preprocessing step responsible for filtering genes that could impair DEGs’ inference. For DEGs' inference, DEGRE uses a Generalized Linear Mixed Model (GLMM) with a negative binomial distribution. Due to the limited number of samples, a stringent threshold for DEGs was set with adjusted p-values or q-values < 0.005.

To detect Differential Alternative Splicing Events (DASE) from the RNA-Seq data, the replicate multivariate analysis of transcript splicing (rMATS v.4.1.2)^34^ was employed. This approach identified different alternative splicing events including skipped exon (SE), alternative 5' splice site (A5SS), alternative 3' splice site (A3SS), mutually exclusive exons (MXE), and retained intron (RI) events. A significance threshold for alternative splicing events was set with a false discovery rate (FDR) < 0.01.

Combined enrichment analysis of DEGs and DASE genes was conducted using Enrichr software^35–37^, incorporating Reactome (RT), Kyoto Encyclopedia of Genes and Genomes (KEGG), Gene Ontology (GO) Consortium^38–40^ and DisGeNET^41^. Stringent adjusted p-values < 0.05 indicated significant enrichments and only the top 10 significant terms were considered.

### Co-expression modules in tissue-specific networks and interactome analysis

The HumanBase tool (https://hb.flatironinstitute.org/) was employed to identify coherent gene clusters in blood tissue-specific networks from the DEGs and DASE genes. Genes within a cluster share local network neighborhoods, forming a cohesive, specific functional module with systematic association. The approach is based on shared k-nearest-neighbors (SKNN) and the Louvain community-finding algorithm. It can mitigate the impact of highly connected genes and highlight the local network structure by establishing connections between genes that are likely to be functionally clustered.

Additionally, data from the Biological General Repository for Interaction Datasets—BioGrid^42^ concerning curated protein and genetic interactions from humans were used to construct an interaction network from the DEGs and DASE genes. Then, information about interactions with SARS-CoV-2 viral proteins, obtained from the BioGRID COVID-19 Coronavirus Curation Project, was integrated into the network. The weights of the nodes were calculated by adding the inverse of the log2 Fold-Change value (1/log2FC). The software Gephi 0.9^43^ was employed to visualize interactions.

### Variant calling and allelic imbalance analysis

In the context of analyses pertaining to expressed SNVs (eSNVs) and insertions/deletions (indels), an allelic imbalance analysis was conducted based on a computational pipeline, PipASE, designed for the detection of Allele-Specific Expression (ASE) within transcriptome data^44^. Initially, the sequencing quality parameters were assessed for each FASTQ file using FastQC (https://www.bioinformatics.babraham.ac.uk/projects/fastqc/). Next, bad-formed reads were removed using Trimmomatic^45^. The filtered reads were aligned to the human GRCh38.p14 reference genome assembly using STAR v3.7 software^31^. Subsequently, the mapped sequences underwent additional processing using SAMtools, which involved sorting, indexing, and read selection based on mapping quality parameters (MAPQ ≥ 30) in BAM files^46^. Then, we masked duplicate reads and performed variant calling in RNA-seq data using MarkedDuplicates and HaplotypeCaller from GATK v4.1, respectively^47,48^. We used ASEReadCounter to determine the read counts for reference and alternative alleles in each position^49^.

The differential expression of genetic variants across the human genome was calculated by the reference allele ratio (ref ratio) in each sample using the following equation: ref ratio = (# of reads with the reference allele)/(# of reads with the reference allele + # of reads with the alternative allele). We required coverage of at least twelve reads per variant site for differential ASE analysis.

### Splice site alteration and haplotype identification around the identified eSNVs from DASE genes

In silico analysis was performed to investigate splicing site alterations around the eSNVs found in the DASE genes. The prediction analysis was performed using ESEfinder and NNSplice tools, with corresponding prediction score thresholds and sequence lengths to reach a sensitivity, and specificity ≥ 80%^50,51^. To perform read-based phasing analysis the HapCUT2 was used with default parameters accessing germline WES BAM files and respective VCFs files. The analysis limitation includes the infeasibility of linking distant variants in haplotypes, since Illumina technology generated short read lengths (100–250 bases)^52^.

### Sequence-based *HLA* typing using RNA-seq data

The *HLA* alleles identification was performed directly from RNA-Seq reads in each sequence. First, RNA-Seq reads in fastq format were mapped to the human chromosome 6 (GRCh38.p14) using bowtie2^53^. The mapped sequences were assembled into 200 bp contigs using the TASR tool^54^ and aligned to *HLA* reference sequences using the NCBI BLAST + 2.13.0 package (https://blast.ncbi.nlm.nih.gov/Blast.cgi). The following alignment parameters were used: -b 5 -v 5. The *HLA* reference sequences of classes I and II genotypes were retrieved in fasta format from the IMGT/HLA database. After alignment, the selected sequences were used to predict *HLA* alleles in the HLAminer tool with the default parameters^55^.

### Institutional review board statement

The study was conducted in accordance with and under the approval of the Pedro Ernesto University Hospital Ethical Committee code: CAAE 0135320.0.0000.5259 version, approved on 01 Sept 2021 version 4).

### Informed consent statement

Informed consent was obtained from all subjects involved in the study or their representatives. Written informed consent was obtained from the participants to publish this paper.

## Results

### Cohort data and transcriptome metrics

Between the low- and high-stable groups, no significant difference was noted in gender composition (sex ratio = 0.43 for both groups) and mean age (53.7 ± 13.8 and 43.8 ± 15.03, respectively). Concerning ethnicity, Group 1 (black—10%, caucasian—50%, mixed—40%) and Group 2 (black—0%, caucasian—30%, mixed: 70%) were also similar (Fisher’s exact test—p = 0.5). Two doses of Coronavac accomplished the primary vaccination schedule in six individuals (60%) in Group 1 and five individuals (50%) in Group 2. The remaining individuals in both groups were vaccinated with two doses of Covishield (Table 1).

The analysis of sequencing metrics reveals that 99% of RNA-seq reads were successfully mapped to the reference genome with a high degree of quality. Approximately 2.8% of the sequencing reads exhibited ambiguity in their mappings. In terms of their distribution across genomic regions, exonic reads constituted 47.8% of the total, while intronic reads accounted for 44.4%, and a mere 0.07% of reads were mapped to rRNA sequences. On average, 83.9% of the bases targeted by the probes exhibited coverage by at least 30 reads. On average, a total of 23,326 genes were detected, and the efficiency of expression profiling was quantified at 0.478%. These results collectively demonstrate the effectiveness of the RNA-seq methodology employed in this study for characterizing gene expression patterns (Supplementary Table 1).

### Differentially expressed genes, alternative splicing events, and functional enrichment

Ten DEGs were identified between the two groups, from which six were downregulated (*ACP3*, *BIRC3*, *VNN3*, *SLPI*, *MT-ATP6*, *MT-ND4L*) and four were upregulated (*KTN1*, *PYHIN1*, *WDR82*, *TMED10*) genes in the low-stable group compared to the high-stable group (Supplementary Table 2). No long non-coding RNAs were recognized as DEGs in this comparison.

DASE analysis from the RNA-Seq data identified twenty-six significant genes (*FCRL3*, *NAGK*, *SLMAP*, *RNF4*, *WDFY3*, *LEF1*, *HLA-A*, *HLA-C*, *HLA-B*, *HDDC2*, *ARL4A*, *UBAP2*, *ZMYND11*, *TBC1D10C*, *NCOR2*, *RUBCNL*, *DCAF11*, *SPG11*, *PSTPIP1*, *DNAJA3*, *CIITA*, *MAP3K3*, *AKAP8L*, *RASGRP4*, *CNOT3*, and *HMGN1*). Exon skipping (ES) was the commonest differential splicing event, representing 44.5% of DASE genes, followed by a retained intron (RI) (33.3%), mutually exclusive exons (MXE) (7.4%), alternative 5' splicing (A3SS) (7.4%), and alternative 5' splice site (A5SS) (7.4%) events. The *HLA-B* gene was involved in two simultaneous differential splicing events (ES and A3SS) (Supplementary Fig. 1, Supplementary Table 2).

**Figure 2:**
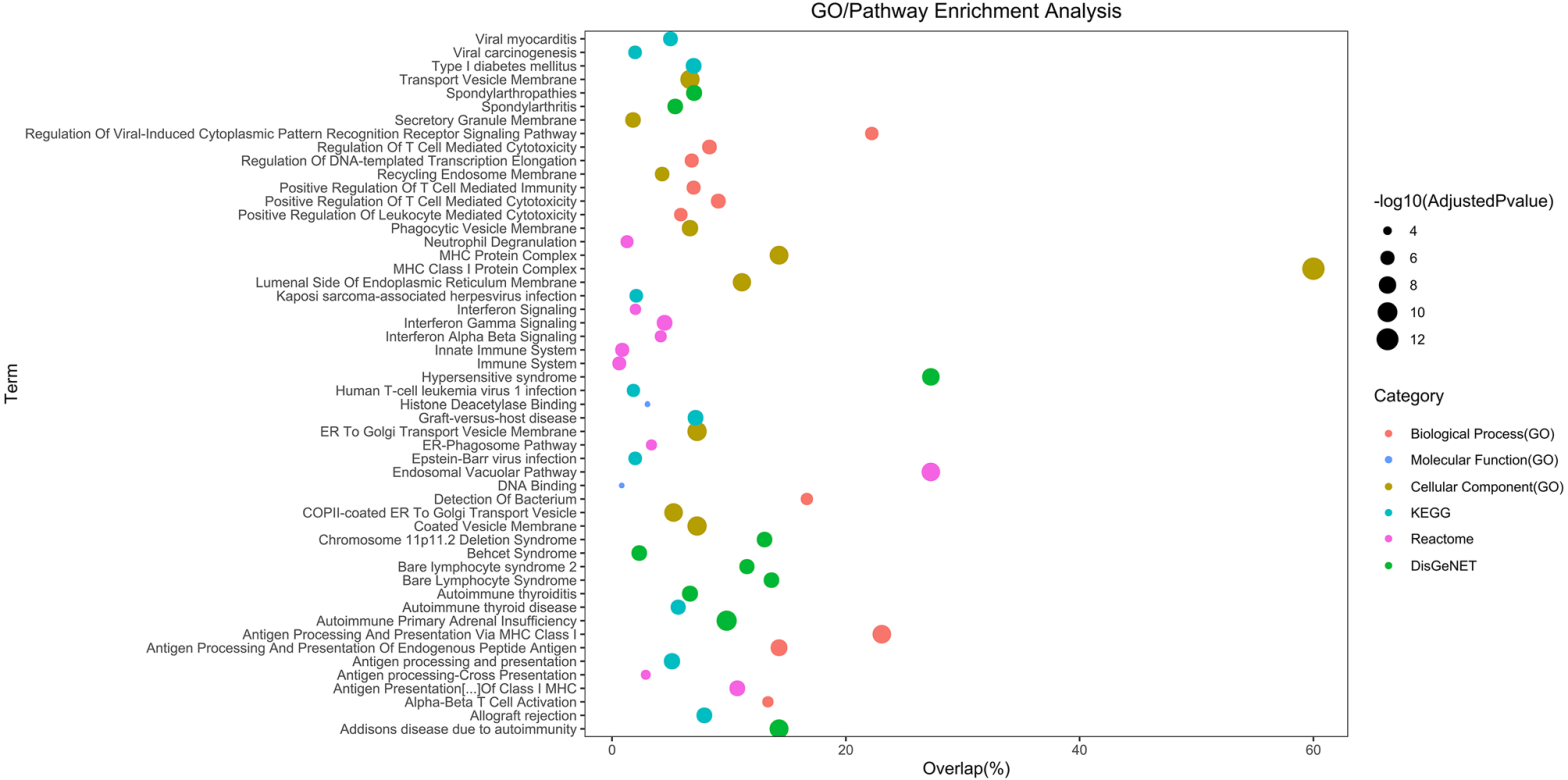
Enrichment analysis conducted on the enrichment of 36 DEGs and DASE genes. The y-axis shows the categories, while the x-axis shows the proportion of genes mapped against the total. Classes are represented by colours and statistical significance by circle size.

Combined functional enrichment analysis of DEGs and DASE genes through Reactome showed significant associations with pathways like endosomal/vacuolar pathway, antigen presentation of class I MHC, interferon-gamma signaling, immune system, innate immune response, neutrophil degranulation, interferon signaling, interferon alpha and beta signaling, and ER-phagosome pathway. In the context of the KEGG database, antigen processing and presentation emerged as the most noteworthy term. Global GO enrichment analysis revealed significant values for all three classes. Enriched Biological Processes (BP) primarily centered around antigen processing and presentation of endogenous peptide antigens via MHC class I. For Molecular Function (MF), enriched terms encompassed histone deacetylase binding and DNA binding. Regarding Cell Component (CC), significant GO terms included MHC class I protein complex and ER to Golgi transport vesicle membrane. Furthermore, disease enrichment analysis using DisGeNET indicated substantial associations with various autoimmune conditions, such as autoimmune primary adrenal insufficiency, Addison's disease due to autoimmunity, hypersensitive syndrome, spondyloarthropathies, and autoimmune thyroiditis (Fig. 2).

### Co-expression modules and SARS-CoV-2 interactome

Analysis of co-expression modules in a blood-specific network through HumanBase revealed one significant module (innate immune response, q value = 0.0022), involving four functionally associated genes: class II major histocompatibility complex transactivator (*CIITA*), Fc receptor-like 3 (*FCRL3*), pyrin and HIN domain family member 1 (*PYHIN1*), and vanin 3 (*VNN3*) (Supplementary Fig. 2). *PYHIN1* and *VNN3* were upregulated and downregulated DEGs, respectively, whereas *CIITA* and *FCRL3* were found to be DASE genes (Supplementary Table 2).

**Figure 3:**
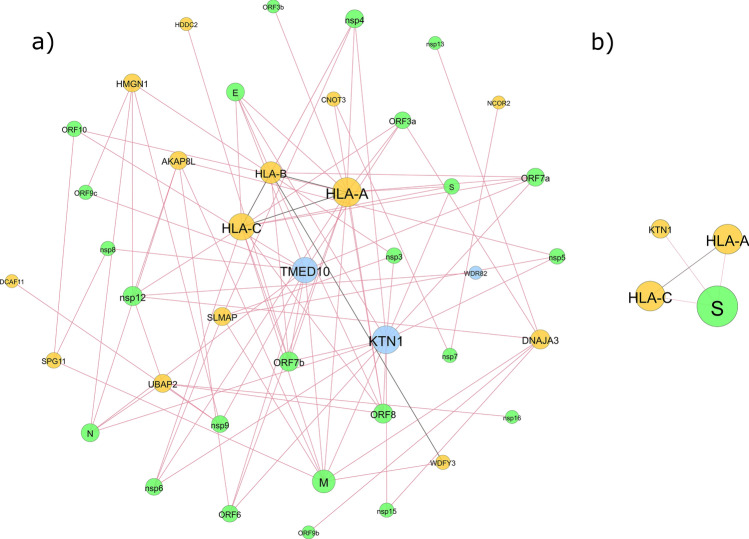
SARS-CoV-2 interactome analysis with DEGs and DASE genes. (**a**) Interactions with all SARS-CoV-2 viral proteins; (**b**) Interactions with SARS-CoV-2 spike protein. The green circles represent genes from SARS-CoV-2, the yellow circles represent DASE genes, and the blue circles indicate the three up-regulated genes. Circle size is proportional to degree (number of interactions), and edge color reflects type: red for interactions between human proteins and SARS-CoV-2, and black for interactions between human proteins.

In the interaction network analysis between human and SARS-CoV-2 proteins, it was observed that out of the 36 proteins encoded by the DEGs and DASE genes, a total of 17 human proteins exhibited robust connectivity with 24 viral proteins, accounting for 88.9% of the entire set of 27 SARS-CoV-2 proteins. Noteworthy interactions in this network were particularly evident for the DASE genes *HLA-A*, *HLA-B*, and *HLA-C*, as well as for the upregulated DEGs *TMED10* and *KTN1*. No interactions were identified for the upregulated DEGs. Moreover, within this group of 17 human proteins, four exhibited interactions amongst themselves: HLA-A, HLA-B, HLA-C, and WDFY3. Amongst the proteins displaying specific interactions with the SARS-CoV-2 spike protein, which is the basis for the mRNA BNT162b2 vaccine, three proteins were retrieved (HLA-A, HLA-C, and KTN1), with direct interactions between HLA-A and HLA-C (Fig. 3).

### Variants in differentially spliced genes and allelic imbalance

**Figure 4:**
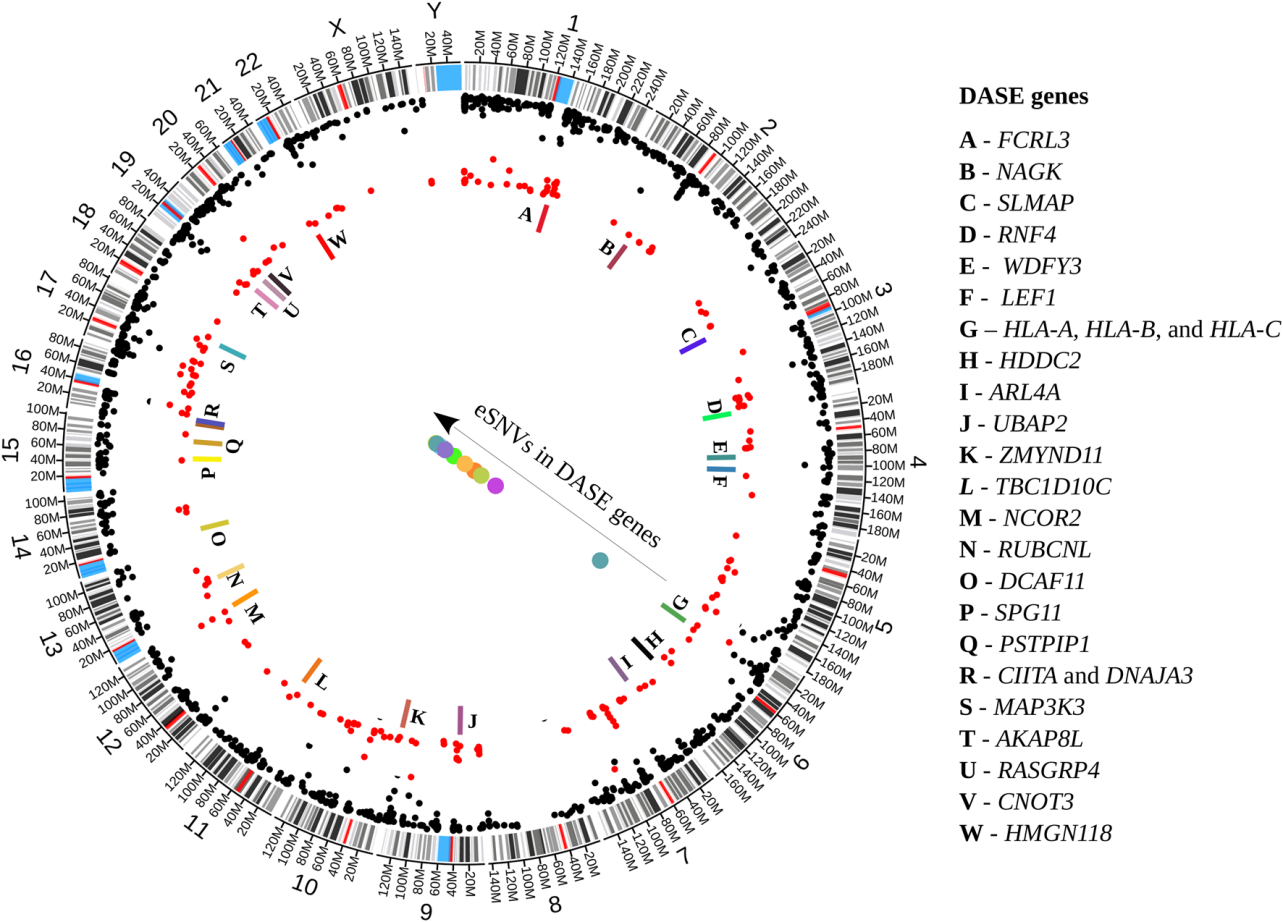
Genomic distribution of eSNVs across the human genome. The outermost layer displays the chromosomal arrangement, followed by the cytoband organization. Within the chromosome layout, all eSNVs with a read count over 12, found in the variant calling step, were represented by black dots. Furthermore, eSNVs shared between the two study groups are represented by red dots. The innermost layer illustrates the distribution of the DASE genes with colored bars. The dots beneath the arrow symbolize the eSNVs located within the DASE genes.

We compared the allelic expression profiles of eSNVs in bulk RNA-Seq data from the two groups. We interrogated 222 eSNVs detected across the groups, with coverage ≥ 12 reads at each site. Only one synonymous variant, NM_001330683.2 (*TTC3*):c.5115G > A p.Lys1705 = , displayed differential ASE (Fig. 4). The genetic variants of the DASE genes were subjected to comprehensive analysis, leading to the identification of twelve eSNVs distributed across the exons 3, 4, 5, and 9 of the *HLA-A* gene and exons 2, 3, 4, and 8 of the *HLA-B* gene (Supplementary Table 3). Group 1, harbored 58.3% (n = 7) of all eSNVs in DASE genes and group 2 showed the smallest amount of eSNVs in DASE genes with 41.6% (n = 5) (Fig. 4, Supplementary Table 3).

**Figure 5:**
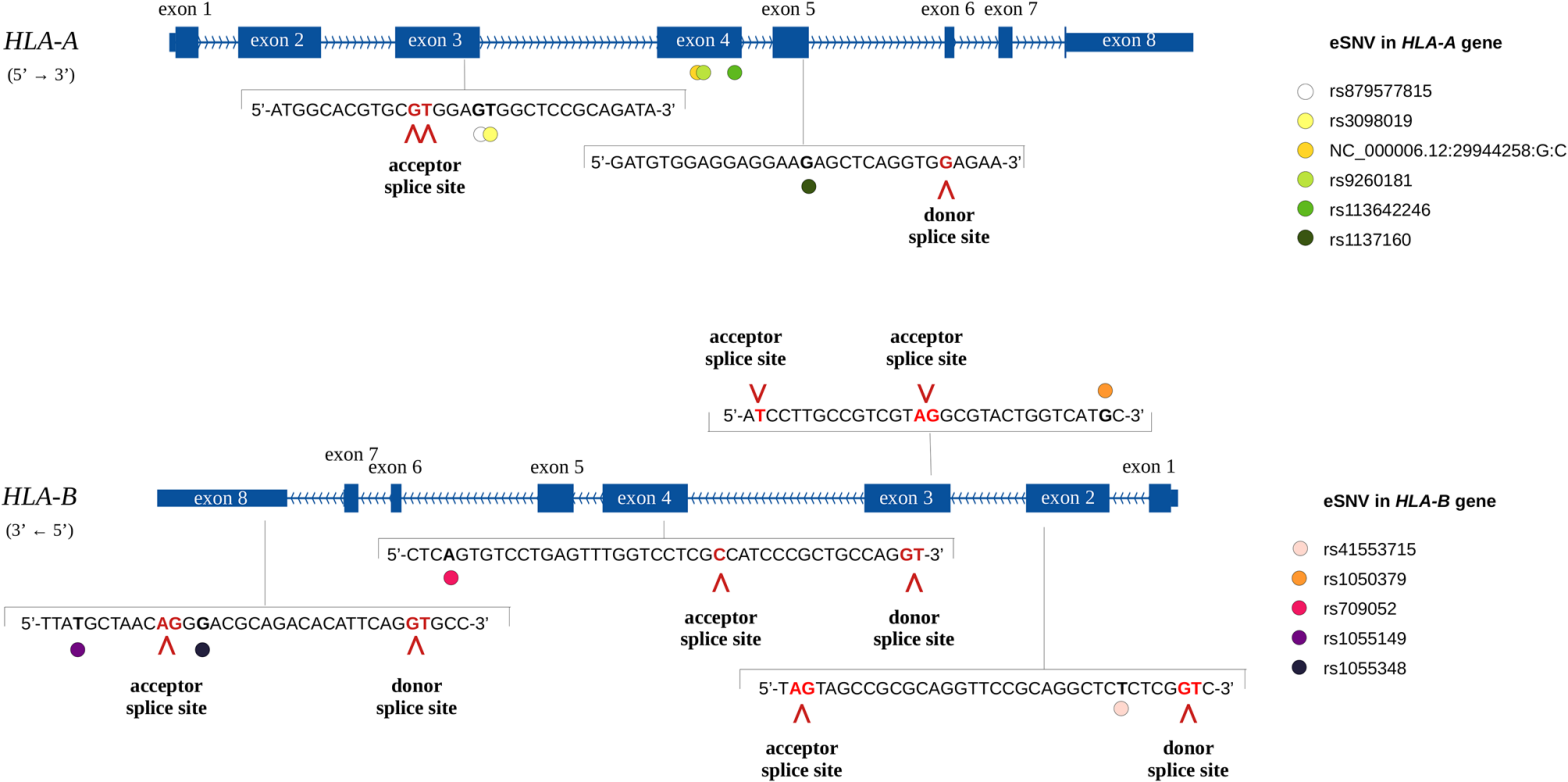
Distribution of the splice site alterations across *HLA-A* and *HLA-B* genes. The structure of *HLA-A* and *HLA-B* genes with exon identification is depicted in dark blue. The sequence context of splice site alterations is visually presented across the genes. eSNVs within the sequence (black bold) are identified by colored dots. Additionally, splice sites (read bold) and their specific alterations are indicated by red arrows.

A splice site prediction analysis of each eSNV of DASE genes revealed a splice site alteration for eight eSNVs (*HLA-A*: rs879577815, rs3098019, rs1137160; *HLA-B*: rs1055149, rs709052, rs1050379, rs41553715, rs1055348) (Fig. 5, Supplementary Table 4). Employing HapCUT2, a read-based phasing analysis was executed to explore additional eSNVs associated with splice site alterations. Two of the aforementioned eSNVs were found to be in phase: the eSNV rs1050379 exhibited phased alignment with rs709055 within exon 3 of the *HLA-B* gene both in a prospective 3′ acceptor splice site, and rs1137160 was in phase with rs74408957 in exon 5 of the *HLA-A* gene in a prospective 5′ donor splice site (Supplementary Table 5).

### *HLA* alleles associated with vaccine response

The *HLA* typing analysis revealed a wide range of *HLA* alleles across the studied groups. The *HLA* class I alleles HLA-A*02:01 and HLA-B*40:01, related to high COVID-19 vaccine response, were found, as well as HLA-A*03:01, HLA-B*08:01 HLA-B*18:01, and HLA-C*07:01 related to low vaccine response. The *HLA* class II alleles predominantly found were HLA-DQB1*06:02 and HLA-DRB1*07:01, both associated with high vaccine response. In addition, two haplotypes previously related to vaccination were identified HLA-DRB1*01:01-DQA1*01:01-DQB1*05:01 and HLA-DRB1*15:01-DQA1*01:02-DQB1*06:02 linked to low and high COVID-19 vaccination response respectively (Supplementary Table 6).

## Discussion

Our knowledge about COVID-19 vaccine-induced immunity and host response immune variability is still limited compared to the ongoing understanding of immunity developed in response to natural SARS-CoV-2 infection. Particularly, COVID-19 mRNA vaccines obtained approval for human emergence use recently and it remains uncertain to what extent these vaccines elicit immune responses or interindividual variability that are either similar to or different from those activated by other vaccine categories, such as inactivated or live-attenuated vaccines. The utilization of high-throughput RNA-based transcriptome analysis to investigate vaccine response and the variability in immune reactions emerges as an exceptionally well-suited tool for uncovering the dynamics of immune responses and gene regulatory networks. Identifying expression changes, differential splicing events, impact variants, and associated biological pathways can offer insights into the interplay between intrinsic genetic/epigenetic background and the extrinsic variable environmental factors. Thus, transcriptome profiling of the host immune response to vaccines is crucial for rationalizing efficient vaccination strategies.

In this study, we used serum antibody detection combined with whole blood RNA-based transcriptome analysis to investigate variability in vaccine response in healthy recipients of a booster (third) dose schedule of the mRNA BNT162b2 vaccine against COVID-19. Notably, the studies concerning host vaccine response to different viruses are conducted with participants of Caucasian descent and such limited ethnic diversity constrains the extrapolation to other populations. To the best of our knowledge, this is the first comprehensive transcriptome study regarding vaccine response to SARS-CoV2 from a mixed population of Latin America. Furthermore, differential splicing events were not previously explored in the context of immune vaccine response against SARS-CoV-2.

Stringent RNA-seq data analysis of the vaccine response to mRNA BNT162b2 booster revealed subtle changes in gene expression, with ten differentially expressed genes between low and high-stable groups. However, DASE analysis identified, for the first time, that distinct differential splicing events mainly regarding *HLA* genes can consistently contribute to variability in vaccine-induced cellular immunity. DEGs and DASE genes were enriched for immune pathways (e.g. antigen presentation of class I MHC, interferon-gamma signaling, innate immune response, neutrophil degranulation, and interferon signaling) and autoimmune diseases (Supplementary Table 2, Fig. 2). Interestingly, two mitochondrial genes (*MT-ATP6* and *MT-ND4L*) are overrepresented among the DEGs. Mitochondrial metabolism is crucial in regulating a broad spectrum of immunological functions. These functions include the differentiation of T cells, the polarization of macrophages, and the response of the immune system against tumors. However, it remains unclear how mitochondrial reactive oxygen species (mROS) and metabolites that originate from mitochondria exert control over immunity^56^.

The significant co-expression module in a blood-specific network ratified the role of *CIITA, FCRL3, PYHIN1,* and *VNN3* genes in innate immune response (Supplementary Fig. 2). *CIITA* gene is a class II major histocompatibility complex transactivator. The ability of an individual to mount an immune response that results in antibody production depends on HLA Class II molecules. SARS-CoV-2 in silico binding affinity to such molecules was demonstrated to predict vaccine effectiveness across Variants Of Concern^57^. Moreover, interindividual variability in antibody responses against SARS-CoV-2 spike protein (ChAdOx1 nCov-19 vaccine) and its receptor-binding domain after the first vaccination showed genome-wide significant association with MHC class II alleles^58^. *FCRL3* gene encodes a member of the immunoglobulin receptor superfamily and is one of the Fc receptor-like glycoproteins genes clustered on the long arm of chromosome 1. Its protein possesses immunoreceptor-tyrosine activation motifs and immunoreceptor-tyrosine inhibitory motifs in its cytoplasmic domain and might act to regulate the immune system^59^. *FCRL3* pathogenic variants are related to different autoimmune diseases, such as rheumatoid arthritis^60^. PYHIN1 is a member of the HIN-200 family of interferon-inducible proteins, characterized by a 200-amino acid motif at their C-termini. Its role encompasses controlling both adaptive and innate immunity, through modulating cytokine production, macrophage, and T cell function, as well as the transcription of a specific target gene^61^. Finally, *VNN3* is a pseudogene that belongs to the vanin family and is responsible for producing an ectoenzyme with pantetheinase activity. The vanin gene family has established roles in oxidative stress and inflammation^62^. Recently, *VNN3* was found to integrate an 11 immune-related gene signature as a biomarker for acute myocardial infarction^63^.

In the interaction network analysis between human and SARS-CoV-2 proteins, a great proportion (88.9%) of the viral proteins interact with 17 out of the 36 genes found to be DEGs or DASE genes. Significant connections within this network were especially noticeable concerning the DASE genes *HLA-A*, *HLA-B*, and *HLA-C*, as well as the upregulated *TMED10* and *KTN1* DEGs. Interestingly, according to Gene Ontology, *TMED10* and *KTN1* are not directly related to immune responses, but their products interact with SARS-CoV-2 proteins, suggesting a role in the viral infection. Research into protein–protein interactions has revealed that SARS-CoV-2 proteins can interact with distinct host genes, disrupting crucial cellular processes, including splicing, translation, and trafficking. As a result, essential biological pathways like the interferon response, which plays a pivotal role in countering viral infections, are suppressed^64^.

Taken together, almost all differential mechanisms found between the low-stable and high-stable groups, regarding DEGs, DASE genes, variants analysis, allelic imbalance, and SARS-CoV-2 network analyses are involved in immune responses, mainly innate immune response. Previous transcriptome analyses for elucidating vaccine effectiveness for different viruses, such as influenza^65^, Hantavax^66^, and VSV-EBOV^67^, have effectively illuminated the intricate patterns of the host's immune response following vaccination. In the context of inactivated influenza vaccines, DEGs at different times of post-vaccination were linked to the IL-17 signaling pathway and oxidative phosphorylation^65^. The Hantavax vaccine, on the other hand, exhibited significant upregulation of DEGs associated with innate immunity and cytokine pathways following vaccination^66^. For VSV-EBOV, transcriptional response displayed characteristics of both innate antiviral immunity and B cell activation^67^.

SARS-CoV-2 vaccines induce widespread immune responses, in both innate and adaptive systems, with extensive crosstalk between them^68–71^. Various pathways within the innate immune system were identified for COVID-19-inactivated vaccines, including the TNF signaling pathway, IL-17 signaling pathway, interactions between viral proteins and cytokines, cytokine-receptor interactions, NF-kappa B signaling pathway, complement and coagulation cascades, B-cell receptor signaling, and Toll-like receptor signaling pathway^72,73^. Previous research on the BNT162b2 mRNA vaccine revealed that it not only triggers a robust innate immune response, including pathways like the Toll-like receptor signaling pathway but also induces epigenetic reprogramming of myeloid cells^68^. Furthermore, BNT162b2 booster vaccination stimulates an increased innate immune response compared to primary vaccination, which is evidenced by a greater frequency of CD14 + CD16 + inflammatory monocytes, a higher concentration of plasma IFNγ, and a transcriptional signature of innate immunity^68^. It demonstrates the ability of mRNA vaccines to prepare the innate immune system for a more robust response after booster immunization. Transcriptome analysis in the elderly demonstrated the activation of interferon-activated genetic programs after a single booster dose of the BNT162b vaccine 15 months after recovering from COVID-19^74^. Besides, the transcriptome of peripheral blood immune cells was also used to evaluate the impact of prior BNT162b2 vaccination on the innate immune response of hospitalized COVID-19 patients infected with the SARS-CoV-2 Beta variant, revealing an enhanced JAK-STAT-mediated immune response at day 10 in vaccinated individuals, in comparison to unvaccinated ones^75^. Recently, time-series transcriptome analysis (before the first vaccination; day 22 after the second vaccination; days 90 and 180 before the third vaccination; and day 360 post-third vaccination) of peripheral blood mononuclear cells from individuals who received the SARS-CoV-2 mRNA vaccine identified genes with fluctuating expression levels and gradual increasing in expression levels from the time before vaccination to the day 360 after a booster dose, as well as genes with increased expression levels at day 360 alone. Besides, pathway enrichment analysis indicated that consistently upregulated genes were associated with immune system signaling, including pathways like interferon signaling and cytokine signaling. Conversely, genes that were consistently downregulated showed a more general connection to terms like ribosomal proteins and translation but were still linked to T-cell functions^76^. Investigations into COVID-19 vaccines and patients infected with COVID-19 have also indicated enriched pathways related to oxidative phosphorylation, ribosomes, and certain human conditions^68,77,78^. However, further investigation is required to determine whether the gene expression within these pathways is linked to the effects of the vaccine.

Notably, *HLA* alleles have been linked to differential responses to vaccination^79^, and immune responses to viral vaccines are impacted by genetic variants within the *HLA* genes^80^. *HLA* genes play a prominent role in activating and regulating the immune system, establishing the mechanism by which processed antigenic epitopes are presented to T cells. Thus, the potential to initiate an immune response to a vaccine is partially governed by the immune response genes found within the *HLA* complex. The *HLA* genes are categorized into class I genes (A, B, and C), class II genes (DR, DP, and DQ), non-classical genes positioned within the class II cluster (TAP and DM), and class III genes. Due to the constraint of HLA-restricted antigen recognition and presentation, as well as the ethnic variation in *HLA loci*, different types of vaccines (such as live attenuated, inactivated, peptide-based, mRNA, and adjuvanted vaccines) might not uniformly exhibit effectiveness across populations.

Like other viruses, the genetic diversity of HLA molecules can also impact the incidence, susceptibility, and severity of COVID-19, as well as the host response variability^81,82^. Curiously, an underlying genetic factor that could potentially contribute to the reduced frequency of SARS-CoV-2 cases in Africa is the presence of distinct *HLA* alleles within the African population, when contrasted with other geographical areas. So, it was speculated that populational *HLA* variability could be correlated with COVID-19 incidence^81^.

An effective presentation of viral peptides via *HLA* class I alleles comprises a faster infection clearance, reducing the susceptibility and severity of COVID-19^82^. Possible reasons for the influence of the marked DASE in *HLA* genes over BNT162b2 booster vaccine response in our study may encompass reduced efficacy of specific HLA proteins in binding and displaying SARS-CoV-2 peptides to T lymphocytes compared to others and distinct *HLA* alleles showcasing a diverse range of epitopes. Notably, the *HLA* alleles previously associated with distinct COVID-19 vaccine responses^83–85^ were indeed detected across the different groups we examined. Surprisingly, though, we did not observe a deterministic link between these *HLA* alleles and the categorization of individuals into low-stable or high-stable vaccine response groups. This observation prompts us to consider the complex interplay of factors contributing to vaccine responses in a mixed Brazilian population with a complex genetic landscape, characterized by a wide range of *HLA* alleles and haplotypes^86^.

Among the individuals in group 1, 30% had a prior reported COVID-19 history, and the remaining stated that they had never been diagnosed with COVID-19. In group 2, 40% of the individuals had experienced COVID-19. Since both groups had individuals with a history of COVID-19 that occurred long before the booster (Table 1), the influence of prior natural infection on the vaccination response might be minimal. Although BNT162b2 is a mRNA vaccine designed to encode SARS-CoV-2 spike protein, we cannot fully discard the possibility of a previous exposition to natural SARS-CoV-2, even in individuals of the low-stable group reporting to had no history of previous COVID-19. From December 2021 to August 2022, the highly transmissible Omicron variant rapidly spread worldwide and became the predominant strain circulating in Brazil^87^, causing mild or no symptoms in most of the population (https://outbreak.info/location-reports?xmin=2023-02-14&xmax=2023-08-14&loc=BRA). Moreover, all the individuals in our study had a primary vaccination schedule with a whole inactivated virus vaccine or a modified chimpanzee adenovirus ChAdOx1 as a vector. Hence, we suggest that an insufficient or suboptimal innate immune response during the primary vaccination schedule, attributed to the differential processes elucidated by our study, might influence the immune response to subsequent mRNA-boosting vaccinations.

An aspect that draws significant attention in our study is the involvement of different alternative splicing events affecting *HLA* genes in the variability of the immune response. The regulation of gene expression through post-transcriptional mechanisms, including the process of differential splicing of precursor mRNAs (pre-mRNAs), plays a critical role in governing the cell-type and tissue-specific patterns of immune response gene expression^88,89^. Shifts in intron usage such as exon skipping, the inclusion of alternative 5' exons, the use of alternative 3' splice sites, mutually exclusive exons, and intron retention can amplify the intricacy of gene expression and contribute to distinct functional roles in orchestrating a cohesive immune response^88,89^. Differential splicing is controlled by RNA-binding factors that interact with cis-acting RNA elements, thus impacting the assembly of cellular spliceosomes at neighboring splice sites. This regulation can exhibit variability across various immune cell types^90^. The relative abundance of diverse transcript isoforms is further modulated by the expression levels of trans-acting splicing factors^91^. The process of intron removal from nascent RNA, along with exon ligation facilitated by the spliceosome, plays a pivotal role in dictating splice site preference, influencing the profiles of transcript isoforms and differential gene expression, and governing transcriptional processes^92^. Interestingly, enrichment for molecular function through Gene Ontology retrieved “DNA binding’ as a significant term, and splicing events are essentially governed by DNA and RNA binding factors.

There are some limitations in the current study. Firstly, we based our transcriptome correlations analysis on humoral immunity, not exploring cellular immune response biomarkers. Although serum antibody levels serve as a proxy for gauging the initial vaccine response and most of the studies surrounding vaccine response have focused on humoral immunity^93^, it is known that SARS-CoV-2-specific cell-mediated immune response takes part from the immune repertoire induced by COVID-19 vaccines in parallel to the humoral response. Nonetheless, the relevance of antibody levels is demonstrated through investigations concerning protection and prophylaxis with passive immunization. This is also underscored by studies of interference with vaccination by passive immunity, and by research delving into vaccine efficacy, emphasizing quantitative measures of vaccine response^94^. Moreover, antibody response to SARS-Cov-2 has been usually employed in evaluating vaccine efficacy^95^, and transcriptome analyses can reflect both humoral and cellular immune responses. Finally, the comprehension of virus-specific T-cell immunity prompted by COVID-19 vaccines remains to be elucidated. Assessing cellular parameters after vaccination, in contrast to antibody assays, presents challenges, thereby diminishing the reliability of these measures. We did not access baseline antibody levels before or in the first weeks after boosting vaccination. So, we do not know the intensity and quality of the booster vaccine response.

## Conclusions

In conclusion, collectively our data indicate that RNA-seq-based transcriptome offers a predictive instrument to assess the diversity in immune reactions within genetically heterogeneous healthy populations. Furthermore, the innate immune response plays a pivotal role in COVID-19 vaccination, shaping the adaptive response after boosting. Also, there is a clear relationship between differential splicing events in *HLA* genes and variability in boosting immunity.

### Supplementary Information

Supplementary Legends.

Supplementary Figure 1.

Supplementary Figure 2.

Supplementary Table 1.

Supplementary Table 2.

Supplementary Table 3.

Supplementary Table 4.

Supplementary Table 5.

Supplementary Table 6.

## Data Availability

The datasets used in the study are available online via the Gene Expression Omnibus database under accession number PRJNA1015225 (https://www.ncbi.nlm.nih.gov/sra/?term=PRJNA1015225).
