# Supplementary material for: Immune response stability to the SARS-CoV-2 mRNA vaccine booster is influenced by differential splicing of HLA genes
Source: Sci Rep. 2024 Apr 18;14:8982. doi: 10.1038/s41598-024-59259-1 (PMC11026523; doi:10.1038/s41598-024-59259-1)
Supplement: Supplementary file 1 — Supplementary Legends. [file 41598_2024_59259_MOESM1_ESM.docx]

**Supplementary Information**

**Supplementary Figure 1:** Significative module obtained in the co-expression modules analysis in the blood-specific network through HumanBase.

**Supplementary Figure 2:** Significant differential splicing events observed for the 36 genes. The y-axis shows the number of differential splicing events, while the x-axis shows the different categories of differential splicing. Genes involved in each category are shown within the bars.

**Supplementary Table 1:** Overview of the sequencing metrics.

**Supplementary Table 2:** DEGs and DASE genes found in the study, their chromosome location, and their main related biological pathways.

**Supplementary Table 3:** Expressed single nucleotide variant found in DASE genes.

**Supplementary Table 4:** Splice site predicted scores for eSNVs from DASE genes.

**Supplementary Table 5:** Detailed information on the read-based phase analysis of eSNVs from the DASE genes.

**Supplementary Table 6:** Prediction of class I and II *HLA* alleles from RNA-seq data.
