# Supplementary material for: Immune response stability to the SARS-CoV-2 mRNA vaccine booster is influenced by differential splicing of HLA genes
Source: Sci Rep. 2024 Apr 18;14:8982. doi: 10.1038/s41598-024-59259-1 (PMC11026523; doi:10.1038/s41598-024-59259-1)
Supplement: Supplementary file 3 — Supplementary Figure 2. [file 41598_2024_59259_MOESM3_ESM.pptx]

## Slide 1
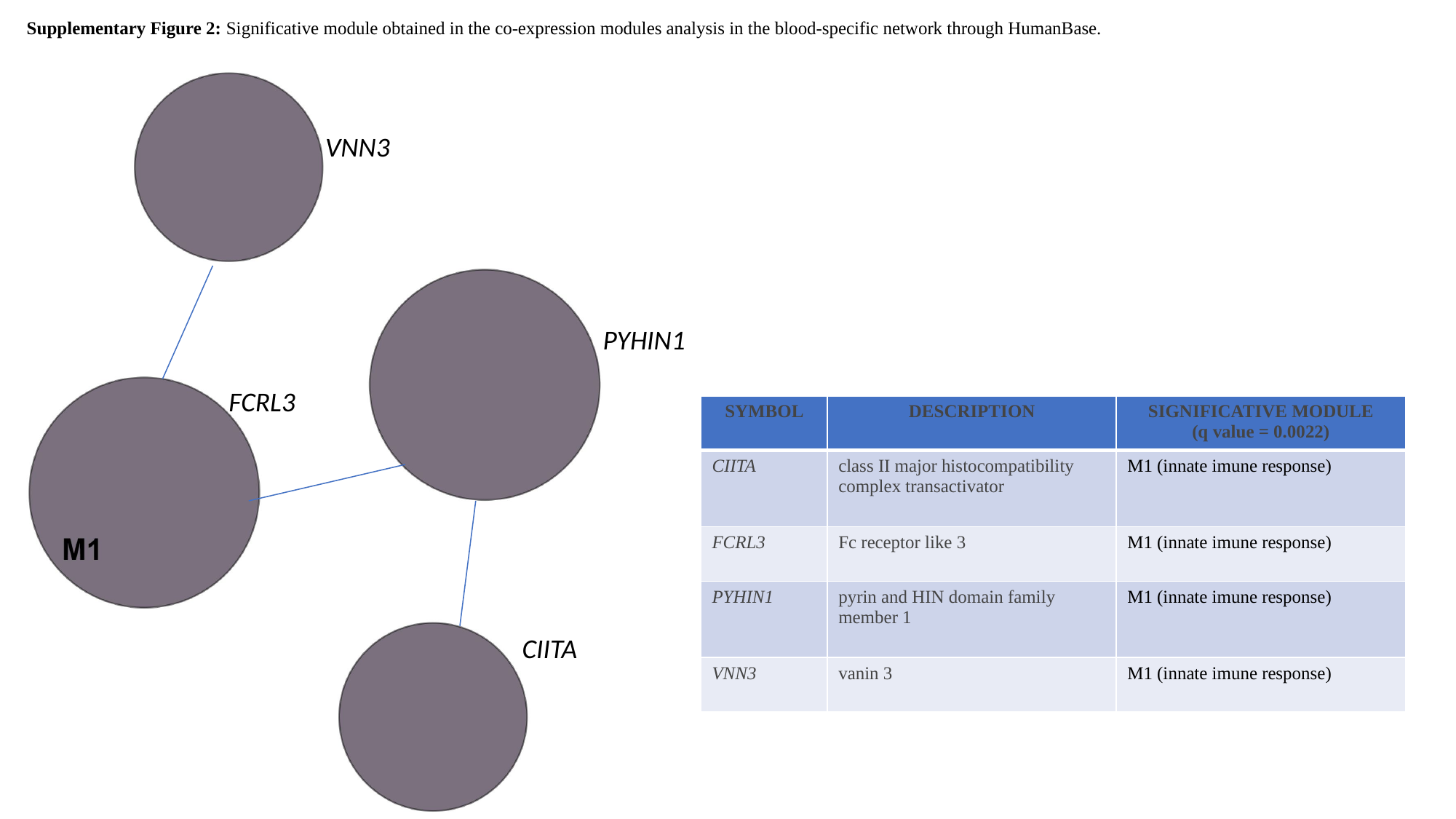

Supplementary Figure 2: Significative module obtained in the co-expression modules analysis in the blood-specific network through HumanBase.
VNN3
PYHIN1
FCRL3
CIITA
| SYMBOL | DESCRIPTION | SIGNIFICATIVE MODULE (q value = 0.0022) |
| --- | --- | --- |
| CIITA | class II major histocompatibility complex transactivator | M1 (innate imune response) |
| FCRL3 | Fc receptor like 3 | M1 (innate imune response) |
| PYHIN1 | pyrin and HIN domain family member 1 | M1 (innate imune response) |
| VNN3 | vanin 3 | M1 (innate imune response) |
