# Supplementary material for: Immune response stability to the SARS-CoV-2 mRNA vaccine booster is influenced by differential splicing of HLA genes
Source: Sci Rep. 2024 Apr 18;14:8982. doi: 10.1038/s41598-024-59259-1 (PMC11026523; doi:10.1038/s41598-024-59259-1)
Supplement: Supplementary file 6 — Supplementary Table 3. [file 41598_2024_59259_MOESM6_ESM.docx]

**Supplementary Table 3:** Expressed single nucleotide variant found in DASE genes.

| **Study group** | **DASE genes** | **Locus** | **Exon position** | **Variant** | **rsID** | **Allelic profile** | **Allele expressed** |
| --- | --- | --- | --- | --- | --- | --- | --- |
| 2 | *HLA-A* | chr6:29943494 | 3 | NM_002116.8:c.570G>A (p.Glu190=) | rs879577815 | imbalance | both |
| 2 | *HLA-A* | chr6:29943495 | 3 | NM_002116.8:c.571T>A (p.Trp191Arg) | rs3098019 | imbalance | both |
| 1 | *HLA-A* | chr6:29944258 | 4 | NM_002116.8:c.756G>C (p.Thr252=) | . | imbalance | both |
| 1 | *HLA-A* | chr6:29944264 | 4 | NM_002116.8:c.762C>T (p.Leu254=) | rs9260181 | imbalance | both |
| 2 | *HLA-A* | chr6:29944309 | 4 | NM_002116.8:c.807G>A (p.Ala269=) | rs199474634 | monoallelic | monoallelic ref |
| 2 | *HLA-A* | chr6:29944609 | 5 | NM_002116.8:c.1005G>C (p.Lys335Asn) | rs1137160 | imbalance | both |
| 1 | *HLA-A* | chr6:29948408 | 9 | NM_002116.8:c.*719G>A | rs113642246 | imbalance | both |
| 2 | *HLA-B* | chr6:31354171 | 8 | NM_005514.8:c.*130A>C | rs1055348 | imbalance | both |
| 1 | *HLA-B* | chr6:31354181 | 8 | NM_005514.8:c.*120C>T | rs1055149 | imbalance | both |
| 1 | *HLA-B* | chr6:31355456 | 4 | NM_005514.8:c.756T>C (p.Thr252=) | rs709052 | imbalance | both |
| 1 | *HLA-B* | chr6:31356377 | 3 | NM_005514.8:c.409C>T (p.His137Tyr) | rs1050379 | monoallelic | monoallelic alt |
| 1 | *HLA-B* | chr6:31356732 | 2 | NM_005514.8:c.299A>T (p.Glu100Val) | rs41553715 | imbalance | both |
