# Supplementary material for: Immune response stability to the SARS-CoV-2 mRNA vaccine booster is influenced by differential splicing of HLA genes
Source: Sci Rep. 2024 Apr 18;14:8982. doi: 10.1038/s41598-024-59259-1 (PMC11026523; doi:10.1038/s41598-024-59259-1)
Supplement: Supplementary file 7 — Supplementary Table 4. [file 41598_2024_59259_MOESM7_ESM.docx]

**Supplementary Table 4:** Splice site predicted scores for eSNVs from DASE genes.

| **chrom** | **position** | **Gene** | **rsID** | **Splice site alteration (score)** | | |
| --- | --- | --- | --- | --- | --- | --- |
|  |  |  |  | **ESEFinder** | **NNSPLICE 0.9** | |
| chr6 | 29943494 | *HLA-A* | rs879577815 | . | Acceptor (0.99) | . |
| chr6 | 29943495 | *HLA-A* |  | . | Acceptor (0.99) | . |
| chr6 | 29944609 | *HLA-A* | rs1137160 | 5’ donor (6.68) | . | . |
| chr6 | 31354171 | *HLA-B* | rs1055348 | 5’ donor (7.42) | Acceptor (0.81) | Donor (0.57) |
| chr6 | 31354181 | *HLA-B* | rs1055149 | . | Acceptor (0.81) | Donor (0.57) |
| chr6 | 31355456 | *HLA-B* | rs709052 | 3′ acceptor (8.03) | . | Donor (0.92) |
| chr6 | 31356377 | *HLA-B* | rs1050379 | 3′ acceptor (8.03) | Acceptor (0.59) | . |
| chr6 | 31356732 | *HLA-B* | rs41553715 | . | Acceptor (0.61) | Donor (0.95) |
|  |  |  |  |  |  |  |
| ESEFinder threshold (acceptor: 6.63/donor: 6.67) | | | | | | |
| NNSplice threshold (acceptor/donor: 0.4) | | | | | | |
