# Supplementary material for: Immune response stability to the SARS-CoV-2 mRNA vaccine booster is influenced by differential splicing of HLA genes
Source: Sci Rep. 2024 Apr 18;14:8982. doi: 10.1038/s41598-024-59259-1 (PMC11026523; doi:10.1038/s41598-024-59259-1)
Supplement: Supplementary file 8 — Supplementary Table 5. [file 41598_2024_59259_MOESM8_ESM.docx]

**Supplementary Table 5:** Detailed information on the read-based phase analysis of eSNVs from the DASE genes.

| **Index^b^** | **Copy A^c^** | **Copy B^d^** | **Chrom** | **Position** | **Ref allele** | **Alt allele** | **VCF genotype field** | **Pruning status^e^** | **Mismatch quality^f^** | **# haplotypes^g^** | **rsID** |
| --- | --- | --- | --- | --- | --- | --- | --- | --- | --- | --- | --- |
| ^a^BLOCK: offset: 68916 len: 2 phased: 2 SPAN: 3 fragments 4 | | | | | | | | | | | |
| 68916 | 0 | 1 | 6 | 31356374 | C | T | 0/1:1,4:5:30:161,0,30 | 0 | . | 100.00 | rs709055 |
| 68917 | 0 | 1 | 6 | 31356377 | G | A | 0/1:1,3:4:33:123,0,33 | 0 | . | 100.00 | rs1050379 |
| ^a^BLOCK: offset: 53155 len: 2 phased: 2 SPAN: 8 fragments 2 | | | | | | | | | | | |
| 53155 | 0 | 1 | 6 | 29944609 | G | C | 0/1:4,2:6:72:72,0,162 | 0 | . | 60.84 | rs1137160 |
| 53156 | 0 | 1 | 6 | 29944617 | G | A | 0/1:3,2:5:75:75,0,120 | 0 | . | 60.84 | rs74408957 |
|  |  |  |  |  |  |  |  |  |  |  |  |
| ^a^BLOCK: offset: <SNV offset> len: <SNV span of block> phased: <# SNVs phased> SPAN: <base pair span of block> fragments <# of fragments in block> | | | | | | | | | | | |
| ^b^VCF file index (1-based index of the line in the input VCF describing variant) | | | | | | | | | | | |
| ^c^allele on haploid chromosome copy A (0 means reference allele, 1 means variant allele, - for an unphased variant) | | | | | | | | | | | |
| ^d^allele on haploid chromosome copy B (0 means reference allele, 1 means variant allele, - for an unphased variant) | | | | | | | | | | | |
| ^e^discrete pruning status (1 means pruned, 0 means phased) | | | | | | | | | | | |
| ^f^mismatch quality: phred-scaled estimated probability that there is a mismatch [single SNV] error at this SNV (0 means SNV is low quality, 100 means SNV is high quality) | | | | | | | | | | | |
| ^g^number of haplotype-informative fragments covering this variant in the input | | | | | | | | | | | |
